# On the Vernacular Language Games of an Antagonistic Online Subculture

**DOI:** 10.3389/fdata.2021.718368

**Published:** 2021-08-10

**Authors:** Stijn Peeters, Marc Tuters, Tom Willaert, Daniël de Zeeuw

**Affiliations:** ^1^ Department of Media Studies, University of Amsterdam, Amsterdam, Netherlands; ^2^ Artificial Intelligence Laboratory, Vrije Universiteit Brussel, Brussels, Belgium

**Keywords:** language games, deep vernacular web, 4chan, encyclopedia dramatica, language communities

## Abstract

In this paper we develop an empirical, big data approach to analyze how alt-right vernacular concepts (such as *kek* and *beta*) were used on the notorious anonymous and ephemeral imageboard 4chan/pol/and the fan wiki Encyclopedia Dramatica. While 4chan/pol/is broadly regarded as an influential source of many of the web’s most successful memes such as Pepe the Frog, Encyclopedia Dramatica functions as a kind of satirical Wikipedia for this meme subculture, written in high concept and highly offensive vernacular style. While the site’s affordances make them distinct, they are connected by a subcultural style and politics that has recently become increasingly connected with violent right-wing activism, forming a loose subcultural language community. Contrary to “memetic” theories of cultural evolution in media studies, our analysis draws on theoretical frameworks from poststructuralist and pragmatist philosophies of language and deploys empirical techniques from corpus linguistics to consider the role of online platforms in shaping these vernacular modes of expression. This approach helps us to identify instances of vernacular innovation within these corpora from 2012-2020—a period during which the white supremacist “alt-right” movement arose online. Through these analyses we contribute both to ongoing interdisciplinary attempts to bridge the gap between cultural-theoretical and computational-linguistic approaches to studying online subcultures, and to the empirical study of the vernacular roots of the “toxic memes” that appear to be an increasingly common feature on social media.

## Introduction

Alongside the growth of misinformation, in recent years social media platforms have faced a problem of “ironic Nazis” or “non-ironic Nazis masquerading as ironic Nazis” who use “comical-meme language” to hide their violent intent behind a veil of “inside jokes and plausible deniability” (Goldenberg and Finkelstein, 2020, p.3). This is not an entirely new phenomenon. Jean-Paul Sartre once remarked Nazis would often “amus(e)” themselves with disparaging jokes confident in the knowledge that their adversaries were “obliged to use words responsibly” [Sartre, 1995 (1949) p.20], thereby weaponizing language games to their advantage. Playing ironic and irresponsible games with language is also a longstanding feature of vernacular Internet communities, who imagine themselves as inhabiting regions of the web that exist outside of normal, real life (Pfaffenberger, 1996; Azriel, 2005; de Zeeuw and Tuters, 2020).

What is new here is the possibility to use big data methods to study how such language is used, where and *when* it developed, and (remarkably) how new language games emerge, both by innovating and canonizing new terms and by transforming the meaning of other terms. The methodological wager in our research is that both *change* and novelty can be gauged from the way the larger contexts of terms’ shifting use over time. In detecting such changes as well as the emergence of new words and phrases we claim to be able to identify aspects of the elusive language games that are so characteristic of this language community. To do so, we leverage Natural Language Processing (NLP) techniques in the analysis of large textual corpora from two websites: the notorious anonymous imageboard 4chan, specifically its far right/pol/(“politically incorrect”) board, and the fan wiki Encyclopedia Dramatica (henceforth ED), in the period 2012-2020.

These *language games* also extend to the reiterated images (or “memes”) that are associated with these communities—the 4chan “imageboard” is often considered as the source of many of the web’s most successful subcultural memes. Studying 4chan memes—and how they “evolve” over time—potentially offers insights into dynamics also at play in social media platforms, which have become plagued with toxic discourse in recent years. Exemplary here is the case of “Pepe the Frog”, a meme that initially became popular on 4chan before exploding into public attention, whose apparent “meaning” shifted over the course of the mid-to-late-2010s from a harmless marker of in-group belonging to a far-right symbol to then in turn become partially recuperated by pro-democracy activists in Hong Kong (Tuters and Hagen, 2020). Such image-memes as Pepe are typically rendered in a simplified style that makes them easy to adapt. However, as slang expressions in the form of text constitute an even more ephemeral form of expression—in that they take even less time and effort to produce—their pace of change should be even more rapid than in the case of image-memes, while the underlying (subcultural) dynamics may be expected to be similar.

While the so-called “alt-right” emerged to widespread public attention in the mid 2010s as a far-right movement that stood out for its non-ironic use of ironic humour Hawley (2017), both ED and 4chan were already established breeding grounds for ironic far right memes. While it has been argued that these fringe online spaces have always been fundamentally bigoted Phillips and Milner (2020) and drowned in “toxic masculinity” Massanari (2017), it has also been alleged that this is a more recent “reactionary turn” congruent with the rise of the alt-right (Tuters and Hagen, 2020). With these hypotheses in mind, the paper proposes to explore the changing contexts of language-use on these two sites, asking the question: can we observe meaning change and vernacular innovation in the data, how can this innovation be characterized, and do these changes and innovations confirm the idea of a “reactionary turn” taking place in these online spaces? If, as discussed in section two below, we can understand language as characterized by a constant tension between change and stasis Deleuze and Guattari (1987), then the study below focuses on communities associated with the vernacular end of that spectrum, what we call *subcultural language communities*, whose speech acts are furthermore governed by the (often arcane) rules of local language games.

The communities studied in this article see themselves as subcultural, insofar as they are opposed to a “mainstream” oftentimes associated with the corporate web of social media as well as progressive liberal culture more generally—the latter which accounts for their “reactionary” status. While subcultures are always rebellious, they can also contain “the seeds of a more radical dissent” (Brake, 2013). In recent years the 4chan imageboard, long central to this subculture, would indeed become indelibly associated with the rise of the white supremacist alt-right ideology particularly and implicated in numerous acts of real world violence (Hawley, 2017). Activity related to this is particularly concentrated on/pol/, one of 4chan’s many forums;/pol/stands for “politically incorrect” and contains the most overtly political activity on the site. For example, the perpetrator of the terror attack on a mosque in Christchurch, New Zealand in March 2019, killing 51 people, posted a manifesto rife with subcultural slang typically found on 4chan and 8chan. He also decorated his rifle with memetic slurs found on the site (Bart, 2021). Considering the politically laden aspect of the scrutinized terms, the paper thus also seeks to contribute to the (empirical) study of cultural conflict around which the current special issue revolves. It should be noted here that our analysis of the relationship between these subcultures and reactionary or far-right (and extreme-right) politics focuses on the Western context, as the user-base of both 4chan and ED is largely Anglo-American, white, male and relatively young (Malmgren, 2017).

In dialogue with theoretical insights from cultural language evolution, this paper therefore explores how tracing the changing meaning of vernacular terms may provide new insight into the dynamics of subcultural language communities online. Due in part to the rapid tempo and general ephemerality of a site such as 4chan, we expect to find rapid change in such a community’ language use—here we define a “language community” as a group of people sharing norms and expectations about how to use language. After tracing our shift in approach from memetics to language games, the next section introduces our two sites of study, 4chan/pol/and ED, after which we outline our method and corpora. The subsequent sections present the results, discussion and conclusion, in which we discuss particularly prominent examples of innovative language use and how this use can be characterized and categorized. Through these analyses we hope to contribute to ongoing interdisciplinary attempts to bridge the gap between cultural-theoretical and computational-linguistic approaches to studying online subcultures.

## From Memetics to Vernacular Language Games

Prior to its association with the emergence of the alt-right, 4chan long had a reputation as the source of many of the most successful “Internet memes” that would then be adopted and spread by users on other platforms; earlier work by Bernstein et al. on 4chan’s/b/board indeed describes the site as an “excellent venue” for studying “innovation diffusion” (2011, p.56). 4chan users often see themselves as the creators of OC (original content) that travels from social media platforms and ends up in mainstream discourse. As such the subculture is constantly subject to processes of recuperation by more mainstream social media sites that we have elsewhere referred to as “normiefication” (de Zeeuw et al., 2020).

While popular understandings of Internet memes typically treat them as a genre of user-generated shared images, media studies literature on the concept has been applied more broadly to other types of content as well, including words and phrases (Shifman, 2013; Milner, 2016). Although this more recent literature is critical of the origin of the term “meme” in ethology, it shares the central conceptual premise of *memetics*, namely that memes *adapt* and *evolve* as they spread across environments. In the context of big data analysis, this conceptual framework lends itself to the study of tracing memes across platforms. Our own previous work has implemented methods for tracing vernacular terms that originated on 4chan before seemingly “spreading” into mainstream social media -- e.g. the Pizzagate conspiracy theory Tuters et al. (2018) and the fictional QAnon persona (de Zeeuw et al., 2020).

In the field of the humanities, the meme is justifiably referred to as a “conceptual troublemaker” in part for how it implies a diminished role for human agency (Jenkins et al., 2013; Shifman, 2013). Moreover, memetics has been critiqued as “dismissing the medium […] as an inert channel” Sampson (2012), meaning that it lacks an account of the role of media environments in co-shaping the evolution of ideas. The critique that memetics reduces human cultural dynamics to universal laws of evolutionary biology has mostly been geared to Richard Dawkins (1976), who coined the term “meme” from an evolutionary biological perspective, and subsequent literature like Susan Blackmore (1999). At the same time, the concept of the meme holds methodological and empirical promise for internet research (Boullier, 2018).

Especially considering the prominence of “circulation” and “spreadability” in shaping cultural dynamics and conflict online Jenkins et al. (2013), Wark and Wark (2019), the idea of the meme prompts a much-needed empirical and analytical shift from the semiotic dimension of cultural objects to their diffusion and refraction in technological networks. Several studies have taken up this task. With their “meme tracker”, Leskovec et al. (2009) traced short phrases to provide insights into the temporality of the online news cycle. He et al. (2016) traced the propagation of memes connected to pro-vaccine and anti-vaccine sentiments, using them to trace the prevalence and rhetorical tactics of different political positions. While methodologically impressive, such studies have not significantly dealt with the aforementioned criticism of memetics, i.e. the dismissal of the medium. As such, this paper seeks to remedy this analytical gap by studying the circulation of imitated objects while also putting the mechanisms of the medium - and how these may affect what a community such as the one we study here focuses on - central.

Furthermore, this approach benefits from the opportunities created by large textual corpora of computer-mediated communication to empirically observe and study aspects of language evolution and innovation. As such, our data-driven approach can be situated against the background of a number of active areas of research. Firstly, this concerns work from the field of complex systems that relies on data from digital information networks such as social media and email to test theoretical assumptions and hypotheses about the propagation of cultural phenomena Heylighen and Chielens (2009), Gleick (2011), Brewer (2016), as well as pioneering studies of memetic phenomena based on electronic chain letters (Goodenough and Dawkins, 1994; Bennett et al., 2003; Chielens, 2003). A second relevant area of work concerns the construction of methods and datasets to empirically investigate the spread and propagation of terms across platforms and communities (e.g. Leskovec et al., 2009; Willaert et al., 2021). Finally, the present article can also be related to previous work in the field of linguistics that uses corpus data from social media to study lexical emergence and innovation (see e.g. Grieve et al., 2017; Grieve et al., 2018). In relation to these areas, our main focus is on the intra-platform dynamics of vernacular innovation, that is, the language games that develop within the context of a single platform marked by specific affordances.

In recent years, alt-right actors have created a vernacular that spread at an unprecedented speed and scale (Squirrell and Sonnad, 2017). In approaching this antagonistic online subculture from a big data perspective, we suggest that it might be conceptualized as “cultural language community” Steels (2012) engaged in what we refer to as *robust vernacular innovation*: recurring and widespread play with semantics that can be seen to constantly change and give new meaning to words, and often has some measure of staying power in that phrases become a significant lasting part of the community’s vernacular*.* In pioneering work on the subject, the computational linguist Luc Steels (2012) developed a theory of cultural evolution as a relational process between agents and their environments by modelling the origins and evolution of language through experiments with artificial agents. Conceptually, Steels’ approach recalls linguistic pragmatics, which considers word meanings as a function of their use as governed by the sets of rules within localized language games (Wittgenstein, 2010 [1953]: 80). According to pragmatics, meanings are thus derived from immediate context and past experiences, which shift from context to context.

Rather than the Darwinian framework presupposed by Dawkins’ notion of the “selfish gene” that forms the conceptual basis of the meme, Steels posits *a co-evolutionary relationship between agents and their contexts* that they inhabit and build—a form of self-organization known as “stigmergy” (Steels, 2012). From a media studies perspective, we can conceptualize these “contexts” as a combination of factors that include both the technical design of websites and their governing socio-cultural norms. In media studies, the preferred terminology for this relational concept is *affordance*. While the concept was initially developed in the field of environmental psychology—to refer to “an invariant variable that is commensurate with the body of the observer” Gibson (2013) — new media are distinguished by the dynamism and malleability of their environments which “adapt their surfaces to their users.” (Bucher and Helmond, 2017). As a relational concept, affordances can also themselves be incorporated into vernacular language games, in the form of folk theories about how technology works (McVeigh-Schultz and Baym, 2015). Anthropological literature has similarly pointed to how “media ideologies,” or the ideas on how a *media channel* should be used, can cohere with and co-structure “language ideologies,” or the ideas on how *language* should be used (Gershon, 2010). Therefore, a material component would seem indispensable to any analysis concerned with the development and spread of memetic objects.

In their “postulate on linguistics”, the philosophers Gilles Deleuze and Felix Guattari (1987) arrive at a similar insight to Steels, although from a quite different departure point. According to their postulate, language is marked by two tendencies: towards stasis and change. Pure change would not constitute language as it would not be recognizable by a community of other speakers—as Wittgenstein noted, by definition a “private language” can not exist (2010 [1953], § 271). Pure stasis on the other hand considers only that which remains the same as language relegating the rest to an extra-linguistic realm. Like Steels, Deleuze and Guattari align their approach with linguistic pragmatics in part by differentiating themselves from Noam Chomsky’s hypothesis of linguistic deep structure—which posits a universal grammatical mechanism in the human mind. In distinction to the latter this approach to language is not meant to be generalized into grand theory of language, but is rather intended to provide conceptual tools by which to describe the conventions of local language games as they are encoded in what Deleuze and Guattari refer to as *order words*.

In Deleuze and Guattari’s conception, fluency or felicity in a given vernacular (or what they call a “minor language”) is negotiated in dialogue with other speakers, and shaped by the milieu within which it takes place. The language that they they use to describe these processes seems quite structural: “There is no individual enunciation[...] not even a subject of enunciation [since] both depend on the nature and transmission of order-words in a given social field” (Deleuze and Guattari, 1987). While they may share an anti-humanist outlook with structuralism—and memetics for that matter—they depart from the former insofar as they consider language to be inseparable from action, and insight derived from the linguist (Austin, 1975). In order to understand these “language games,” it follows that one needs to understand something of the highly dynamic milieus within which they are used and from which their “rules” are often derived.

Drawing on Austin’s notion of the illocutionary speech act—an utterance with a functional effect such as commanding or requesting that something be done—Deleuze and Guattari argue that order words do not however merely concern commands, but rather “every act that is linked to statements by a “social obligation”” or “implicit presupposition” (Deleuze and Guattari, 1987). Order words thus arguably reflect the (unwritten) local rules that govern felicitous speech within a subcultural language community. Such a language theory bypasses the problem of determining statements’ (manifest) meaning—often extremely tricky in the case of Internet discourse rife with irony—by focussing instead on the (latent) work they do in a community whose practices and contentions we can study as being shaped by various affordances.

Since 4chan is an anonymous forum without registered user accounts (by default users post as “Anonymous”), expressions of identity are performed and encapsulated by how their members interact through text and image: their use of a highly idiosyncratic ingroup vernacular. Knowing how to speak like an “anon” (as 4chan users call themselves) thus requires negotiating a variety of laws and axioms that quite literally constrain these utterances like the rules of a game. Extending beyond the boundaries of 4chan, these laws and axioms are often imagined as pertaining to a set of discussion forums and websites that we have referred to collectively as the “deep vernacular web” (de Zeeuw and Tuters, 2020). This category of sites which share a family resemblance extends beyond just 4chan and can be considered to include other image boards, as well as other types of sites such as Encyclopedia Dramatica or parts of Reddit. In accordance with the inherently “unserious” play logic of these subcultural spaces, these language games are often literally understood as games, i.e. as a play on the identity and discourse of the users interacting. Crucially, through this gamification of online interaction and discourse, where online utterances are neither constrained by the truth-value of their propositional contents (or reflecting the speech actor’s actual beliefs), nor verifiable by non-contingent identity cues, language becomes highly volatile and unstable.

4chan is especially prone to what we could call a *living discourse* akin to the modalities of everyday speech rather than literary discourse. This is stimulated by the affordance of not only *anonymity* but *ephemerality*: old or inactive threads on 4chan are deleted as soon as a new one is made, encouraging a rapidly evolving vernacular (Bernstein et al., 2011). Further still, some scholars consider 4chan’s artifacts as a form of “digital folklore, though a degenerated one” that is characterized by an ephemeral temporality and emphasis on antagonism that bears comparison to sensemaking processes associated with preliterate oral cultures (Venturini, 2003)^1^. Sedimented in these vernaculars are the social worlds and cultural imaginaries of their participants, and new users unfamiliar with any community’s social world are typically invited to “lurk moar” — meaning they are to first familiarize themselves with the local cultural habits, dialects, and rules before trying to actively engage with and individually appropriate them.

Rather than lurking on 4chan, however, there is also the option to consult various wikis dedicated to archiving deep vernacular web culture. As one of those wikis central to this paper, ED also serves as a subcultural heritage site that documents subcultural slang associated with anonymous Internet culture. As the wiki format retains the content added to it, ED acts as a performative archive that is accumulative rather than constantly self-rejuvenating, as does 4chan. Just like Wikipedia, although sections may be deleted and changed, the general structure of the article tends to evolve and expand as users engage with it.

Thus, next to 4chan’s ephemeral current of posts, ED offers a semi-stable companion for those wishing to be up-to-date with the latest subcultural vernacular and controversies. ED is an “altpedia”, an online encyclopedia that rejects Wikipedia’s “neutral point of view” policy in favour of a hyper-partisan style of knowledge making (de Keulenaar et al., 2019). Described as a “troll archive” Schwartz (2008), much of what ED preserves is rooted in 4chan. If the sites share a common vernacular, they differ significantly however in terms of user numbers and site design. As an imageboard, 4chan can be contrasted to the format of a wiki, by its affordances of anonymity and ephemerality in which memes and slang are used to negotiate a sense of belonging to an essentially chaotic and disorganized community (Tuters and Hagen, 2020). The two platforms are then positioned here as part of the same language community, differing particularly with regards to their materiality, and thereby a study that comprises both can offer a more comprehensive impression of the community in dialogue with this material aspect.

## Data: 4chan and Encyclopedia Dramatica as Vernacular Milieus

As primarily English language websites ED and 4chan/pol/—the part of 4chan we study here—bear certain similarities. While technically distinct these websites are culturally connected—most visibly by their shared use of subcultural meme iconography. On a linguistic level we can also consider these similarities in line with Wittgenstein’s of the concept of “family resemblances,” which he developed as part of his critique of structural linguistics. Using the paradigmatic example of games allowed him to identify “a complicated network of similarities overlapping and criss-crossing” [2010 (1953)] in which there however is no one common feature. In light of the deep vernacular web hypothesis mentioned above, here we posit these overlapping similarities as being the games played with language on these websites which have the effect of “destabilizing” the English language in similar ways. Insofar as our empirical study reveals comparable patterns of language change and novelty between two different websites, in what follows, we consider felicity in a shared vernacular as a proxy for a shared style, which elsewhere has been referred to as “memetic antagonism” (Tuters and Hagen, 2020).

### 4chan

Because 4chan users are anonymous, no matter how familiar they may be with the community, each time they log on anew they appear to others as a complete stranger. These anonymous posters, or “anons,” can however demonstrate their in-group status through performing their familiarity with, and fluency in, the latest vernacular innovations on the site. Anons have thus developed techniques by which to distinguish members of the subcultural in-group from outsiders. Previous research has shown how anonymous posters on 4chan make use of memes and other forms of subcultural capital, which results in strong and often highly antagonistic group dynamics (Nissenbaum and Shifman, 2017; Tuters and Hagen, 2020); this is perhaps most clear the case on/pol/, 4chans board dedicated to political discussion.

Despite its seeming chaos, in media coverage,/pol/has been framed as a central hub for far-right activity, as a neo-Nazi recruitment zone, as a source of ideological inspiration for violent extremists, and as a significant force in the election of Donald Trump (Beran, 2019; Tuters and Hagen, 2020). While these claims are correct to some extent, in addressing the urgency of these issues they may overlook some of the complex vernacular language dynamics that may also draw users to the site. Specifically they tend to overlook the entwining of language games and technical affordances as constitutive factors in creating an antagonistic subculture whose vernacular innovations has been remarkably successful in influencing cultural conflicts beyond its own confines. Instead of this cultural diffusion, however, our case studies highlight the bizarre and self-referential role of vernaculars *within* this community.

### Encyclopedia Dramatica

Encyclopedia Dramatica started in 2005 as a community forum for discussing “drama” taking place within the LiveJournal blogging community. Since then, ED has had an extremely volatile history, with frequent changes in management and domain names that have resulted in numerous outages and prolonged periods of unavailability^2^. In the 2012-2020 interval we study, the site’s focus had changed from its original interest in the LiveJournal community and is more accurately described as an unofficial archive for the trolling subculture associated with 4chan (Phillips, 2015). ED is a comparatively smaller platform than 4chan, with a smaller user base that has moreover diminished over the course of the last decade; nevertheless, as can be seen in our later discussion of our corpora, the site has over the years accumulated a large number of Wiki pages describing people, concepts, websites and other entities of interest to its community.

The comparatively small amount of activity can be explained through the type of site ED it is; as a wiki, its lack of activity is arguably less significant, as its pages represent an archive of events, people, and other phenomena that need only periodically be updated. While ED looks more or less like Wikipedia, it is written in a high concept vernacular that is likely offensive and confusing to the uninitiated. The formats and conventions of the wiki such as in-links and categorization schemas are often intentionally misused—for example, the most common in-link in the corpus is an otherwise insignificant page on the topic of “Shit,” which is rarely linked to for its actual content, but more so to signify a like of whatever is being linked to the page. This is emblematic of the overall tone of the site, which can be seen as a polar opposite to the famed “Neutral Point of View” of Wikipedia.

### Corpora

**FIGURE 1:**
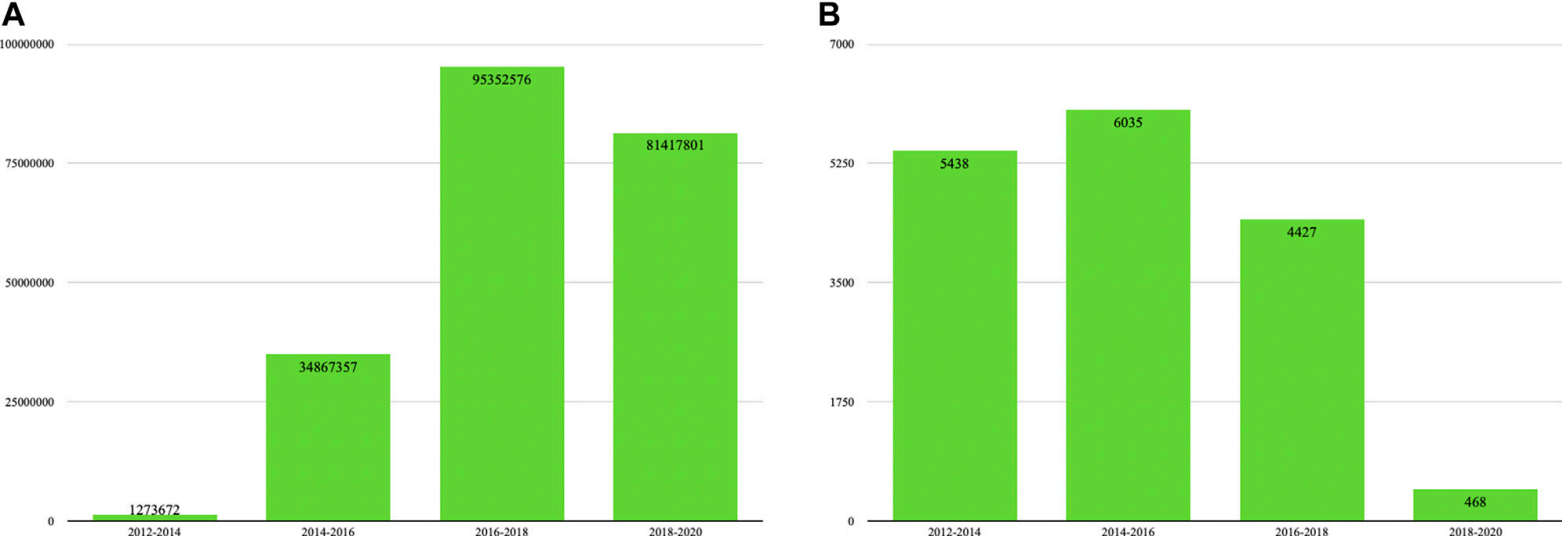
Figure 1**(A)** and Figure 1**(B)**: Items (posts or page revisions) per bin for our 4chan and Encyclopedia Dramatica datasets, respectively.

These two sites then serve as good examples of the type of vernacular language community we are interested in here. Additionally, while both sites make heavy use of images to illustrate their posts and pages, they can nevertheless be said to be predominantly text-based; the bulk of the discourse on both sites occurs in textual form, as the content of a post on 4chan or the content of a page on ED. For our analysis, we therefore collect textual data from the entirety of 4chan’s far-right/pol/forum and ED, covering the period 2012-2020 (see Figure 1).

Data for/pol/is collected using 4CAT, a platform analysis toolkit (Peeters and Hagen, 2018) which contains a dataset comprising 4chan/pol/data going back to 2013. This data is collected continuously by 4CAT itself and, for data prior to 2018, supplemented with archives sourced from 4plebs.org, a third-party 4chan archive that publishes semi-annual “data dumps” on The Internet Archive. These comprise all posts made on various 4chan boards, including/pol/. Both 4pLebs’ and 4CAT’s datasets have been used as “complete” data in other research on 4chan (e.g. Jokubauskaitė and Peeters, 2020; Tuters and Hagen, 2020; Voué et al., 2020). Notably, posts are included in this dataset even if they are later deleted from 4chan (all posts eventually disappear from the site, as forum threads are deleted after a given period of inactivity). The data is then filtered for posts created within the relevant time interval, for a total of 212,911,406 posts, which are subsequently cleaned to remove HTML markup and 4chan-specific markup syntax.

Our dataset for ED is collected using *dumpgenerator*, a software tool for archiving MediaWiki-based wikis published by Archive Team, a loose collective of web archivists affiliated with The Internet Archive. The tool interfaces with the wiki’s own API to download all revisions of all pages on a given MediaWiki-based wiki; this way, one can obtain a dataset that allows the analysis of a given page or set of pages as it was at any moment in the wiki’s history (see e.g. Yang and Nyberg, 2015; Park and Motahari Nezhad, 2018). We collect this data for ED during August 2020, and afterwards discard data not within the main wiki namespace (e.g. user pages, discussions, or pages with image metadata). The final dataset comprises 265,771 revisions (i.e. versions of pages) across 26,305 distinct wiki articles within the relevant time interval. As the collected data contains “wikitext” markup irrelevant for semantic analysis, the data was parsed and cleaned with a custom parser to remove such syntax while retaining as much textual data as possible.

One obvious difference between these datasets is the number of items they comprise; our/pol/dataset contains roughly 800 times as many items as our ED dataset. It should however be noted here that whereas posts on/pol/are typically short - sometimes only a single word - articles on ED are typically far longer. After cleaning, the average/pol/post in our dataset is 261 characters long; the average ED revision on the other hand has a length of 12,290 characters. In other words, if one looks at the amount of data per se rather than the amount of individual items, the difference is less pronounced though still significant, at 55 billion characters (/pol/) versus 3.27 billion characters (ED). Together, the two corpora then provide a solid basis for the identification of innovative language use in this community from two different material perspectives.

## Methods

Other research has analysed the language use of particularly 4chan/pol/with quantitative methods to identify significant language patterns. Much of this research is focused on automated detection of particular categories of hate speech. For example, Hine et al. (2017) use statistical and link analysis to investigate 4chan/pol/demographics, “raiding” of other websites and the prevalence of known hate speech word lists; Gordeev and Potapov (2020) use word embedding models and automated image annotation to measure ‘toxicity’ in a 4chan-based dataset; and Baele et al. (2021) deploy various statistical methods to compare popular topics on six different image boards including 4chan/pol/. Such primarily quantitative approaches are efficient in identifying *what* language is salient, but less so in explaining *why* such language is used and *how* it connects to broader ideologies and concerns. To this end, we combine an initial computational analysis of 4chan/pol/and ED with a qualitative close reading of a number of distinctive terms found through this method.

Ours is therefore a “quali-quantitative” Venturini and Latour (2010) approach situated at the intersections of recent methodological developments in the domains of media theory, “big data” for cultural analysis, and distributional semantics (e.g. van Aggelen et al., 2017; Hamilton et al., 2018). In our case the computational analysis also serves as an initial heuristic and starting point for more qualitative analysis. One alternative approach here could be to create an expert list of relevant language through e.g. a literature review or via the collective domain expertise of the authors. A “bottom-up” computational approach however has benefits in this context; as discussed, language evolves rapidly in these spaces, and knowledge about it may become incomplete quickly, complicating reliance on expert lists. Furthermore, the computational approach on offer here can more readily generalize to other contexts in which domain expertise is less easily available, for example more obscure image boards or emerging online communities.

As our initial step is to computationally identify (order) words that are potentially representative of language games along the lines sketched out in the second section above, we here operationalise these aspects of creative language use as *change* and *novelty,* two factors we can look for within our data using computational methods. Since what we are studying here are subcultural language communities, whose vernacular language use is characterized by its relative rate of change, then the premise is that words that change the most are also the most significant in order to understand our object of study. This initial computational distant reading then serves as a foundation for a subsequent close reading in which we focus on words that represent *robust vernacular innovations*. Following the insights of pragmatics, innovative vernaculars should be marked by changes in words’ contexts of use (as an existing word is appropriated for new purposes), while the invention of hitherto unseen words may also be considered as markers of innovation.

For our initial computational analysis we rank the vocabulary per dataset by usage usage, and retained only the top 5,000 most-used words per corpus. This step ensures that any words marked as significant in the following steps are in fact regularly used within the corpora, and not simply one-off words that might stand out but are otherwise not representative. We then calculated the rate of contextual change for each of these words, to identify words that are “unstable,” being used in different contexts through time, which could signify them being used innovatively. To this end, we first split both corpora in 2-year snapshots, covering the periods 2012-2014, 2014-2016, 2016-2018, and 2018-2020. In the case of the 4chan data, each of these 2-year snapshots starts on January 1, and contains any posts created during the respective period. In the case of ED, the affordances of the platform (a wiki) necessitated a different approach. On a wiki, the same pages are often edited across multiple years, and a given page is a sum of all contributions up to that version, complicating the sorting of a specific page into a given snapshot. Therefore, per ED snapshot, we include the latest *version* of all pages that existed within the respective interval, if that version was made within the interval.

Next, we train a Word2vec model on the case-folded text within each snapshot, using the Continuous Bag of Words (CBOW) architecture as originally suggested by Mikolov et al. (2013),^3^. Mindful of Firth Firth (1957) ’s famous assertion that “you shall know a word by the company it keeps,” Word2vec maps words in a corpus into high-dimensional vector space such that words used in a similar context are closer to each other. For each of the words in the aforementioned top 5,000, we then count the number of *new* words in their 20 most similar words (by cosine distance within the vector space for that snapshot). “New” here means that the word did not occur in the top 20 for the previous snapshot. The amount of new words is then averaged, allowing us to sort words by the *average contextual difference* between snapshots. Words with a higher average difference thus exhibit a more rapidly changing context of use. We then discard all but the top 200 most-changing words per corpus.

Subsequently, we identify “neologisms” in the corpora, that is, words that do not occur in “normal” English and could thus be additional markers of creative language use. To this end, we again use the top 5,000 words, and discard any words that do not occur in the Google Books *English One Million* unigrams word list^4^. As a word list derived from a broad spectrum of English-language books, this can be considered representative of “ordinary” English language, and as such words not occurring on it are interesting in that they are potentially invented by the language community. After combining the lists of neologisms and contextually changing words, and discarding noise (such as proper names) and alternative or colloquial spellings of otherwise ordinary words (e.g. *eachother*), this yields a combined shortlist of 33 words (see Table 1). From this shortlist, three broader categories can be distinguished, which we discuss in the next section.

**TABLE 1:** Words collected from the top 200 changing and new words on both 4chan/pol/and ED, after filtering for misspellings, proper names, duplicates, and other noise. Non-representative order. Words detected by *novelty* marked in yellow; words detected by *change* marked in orange.

Anons	Beta	Burger	Copypasta
Ediots	Evar	faggotry	fantards
Fucktard	furfags	Gamer	jewtube
Kebab	Kek	Lel	libtards
Lulz	Magic	manlet	merchant
Moar	newfag	Pc	pilled
Powerword	raep	redpill	redpilled
Rekt	Shitpost	shitposting	tartlet
Trips	—	—	—

## Results

Our method yields a selection of words pertaining to two types of vernacular innovation on 4chan and ED: completely new word forms (neologisms) and a set of existing words that appear to develop new meanings. On both platforms, the emergence and uses of these terms are governed by local conventions. Based on these conventions, the words can be grouped into three categories: ingroup/outgroup terminology (antagonistic terms referring to the own group or the Other, e.g. *kebab* for muslims), ironic wordplay (deliberate representations of harmful vernacular, e.g. *raep* for rape), and reflexive performativity (words that reflexively signal vernacular language practices themselves, e.g. *shitpost*, *powerword*). Table 2 shows in which category each of the 33 identified words can be placed primarily. In the following sections, we discuss a number of particularly representative examples per category.While there is substantial overlap between these categories, they each offer particular insights into these platforms’ use of vernaculars.

**TABLE 2:** Words collected from the top 200 changing and new words on both 4chan/pol/and ED, after filtering for misspellings, proper names, duplicates, and other noise. Non-representative order. Words primarily categorized as “group terminology” are marked in blue; “ironic word play” in mauve; “reflexive performativity” in khaki.

Anons	Beta	Burger	Copypasta
Ediots	Evar	faggotry	fantards
Fucktard	Furfags	Gamer	jewtube
Kebab	Kek	Lel	libtards
Lulz	Magic	manlet	merchant
Moar	Newfag	pc	pilled
Powerword	Raep	redpill	redpilled
Rekt	Shitpost	shitposting	tartlet
Trips	—	—	—

### Group Terminology

Given the extremely antagonistic nature of the community we investigate, it was unsurprising to find that many of the retrieved vernacular terms on 4chan and ED are used to demarcate and emphasize particular groups of people and identities. Some of these explicitly establish in-groups or outgroups, while others are more ambiguous. Vernacular innovations of in-group terms nevertheless appear to dramatically outweigh innovations of out-group terms. This can be understood as a basic rule of cultural distinction, whereby matters of cultural taste are defined foremost as the distaste of others (Bourdieu 2013 [1984]). The proliferation of this category of vernacular is then consistent with the common characterization of these spaces as politically extremist, marked by a proportionally extreme “distaste of others.”

Both platforms have developed specific terms to refer to themselves - the in-group. On 4chan, the aforementioned term *anon* - like many of the terms discussed in this section detected as a popular “neologism” - is used to refer to oneself or other users; on ED, the term *EDiot* (a portmanteau of ED and idiot) has a similar function. Common vernacular terms were also observed to be used as building blocks for other phrases, so the derogatory suffix *-fag* is is used to refer to novice users as *newfags* on 4chan or as *faggotry* anything at odds with the platform’s cultural ethos, while the suffix *-tard* (derived from “retard”) becomes *fucktards* on ED. In our 4chan dataset, we can identify antagonistic neologisms that are politically oriented (e.g. *libtards* for US liberals or progressives) as well as terms affiliated with the so-called “manosphere,” a men’s right’s movement opposed to feminism and centered on a narrative of reverse victimization (see Ging, 2019). One example we encountered is the term *manlet,* which uses the derogatory diminutive suffix *-let* in reference to a figuratively or literally small man.

That said, it is not always easy to clearly distinguish between in- and out-group terms, in a way that speaks to the highly idiosyncratic and derisive use of language on these platforms. For example, seemingly derogatory out-group terms like *fag* also often applied to oneself or other members of the in-group in a spirit of juvenile self-mockery and ironic endearment. Specifically, this is a characteristic of western male youth cultures, where belonging is paradoxically affirmed by a constant undermining of the communal bond through mutual antagonism, competition, and playful one-upmanship (Walker, 1988). Moreover, as with the terms “punk” and “queer”, the reappropriation of former slurs as markers of in-group identity is a well known dynamic in subculture studies, a tendency that is pushed further to the extreme here and accounts for the ambiguity between being “inside” or “outside” the core group.

One vernacular term for expressing male identity stood out in particular because of its changing context of use: *beta*. As Ging (2019) notes, the beta male identity is closely related to the incel (involuntary celibate) movement that finds a home on various platforms like 4chan and Reddit, and has been connected to misogynistic acts of violence such as the 2018 mass killing in Toronto by a man that identified as a *beta*. This and other similar attacks are in turn referred to on forums like 4chan’s/r9k/(but to a lesser extent also/pol/) as a *beta uprising*. Our analysis shows that in the 2012-2014 and 2014-2016 snapshots of the ED corpus, the term *beta* is used in a similar context as terms referring to computer software and video games (*torrent*, *java*, *skyrim*, *multiplayer*). In the 2016-2018 and 2018-2020 snapshots new uses seem to emerge, where the term *beta* is used in the same context as antagonistic, identitary vernacular terms concerning race and nationality (e.g. *wigger*, *asian*, *britfag*, *irish*, *inbred*), gender and sexuality (*feminazi*, *bisexual*, *virgin*), and online subcultures (*weeaboo*, *goth*, *furfag*). This new usage of *beta* thus signifies the adoption of the concept of the “beta male” as a (sometimes self-deprecating) label. The change in use of this specific term over time in our data reflects the emergence of this new identity formation out of the context of video game and geek culture, which is concordant with scholarly research on “toxic geek masculinity” (Salter and Blodgett, 2017). From its original meaning as a stage of software testing, then, the meaning of *beta* shifted alongside the more comprehensive politicization of online subcultures associated with the rise of the so-called “alt-right”.

**FIGURE 2:**
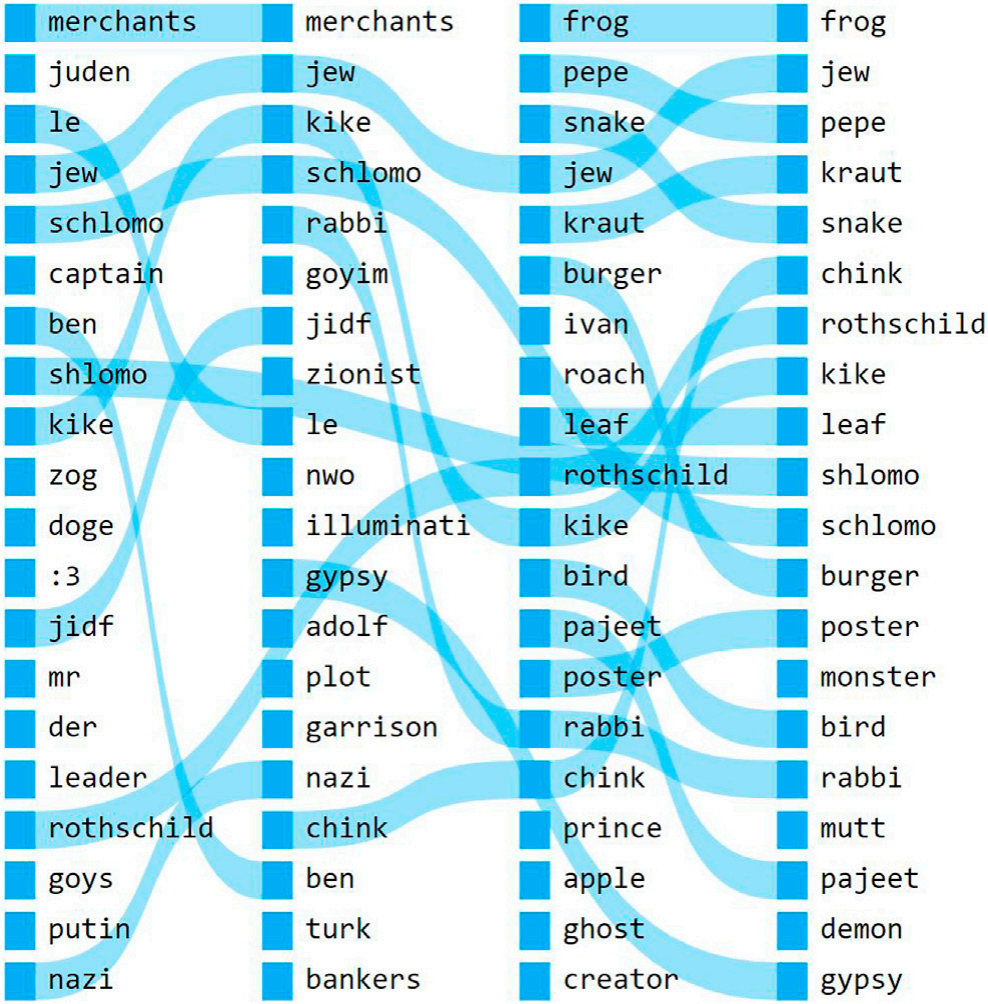
Rankflow diagram of words similar to *merchant* in our 4chan dataset. Similar words are sorted vertically, from most similar to least similar. Each vertical stack represents a single snapshot, respectively 2012-2014, 2014-2016, 2016-2018, and 2018-2020.

In detecting language innovation on these sites we also see a wide variety of neologisms or appropriations of existing words used to express out-group antagonism that reflect racist or otherwise discriminatory attitudes. Examples here are the words *kebab* and *burger*, metonyms for muslims and Americans respectively. Similarly, *merchant* is used on 4chan to refer to people of jewish origin^5^, as well as an expanding range of other antagonized groups—whereas it is initially used in a purely antisemitic context, later its similar words include more diverse ethnic slurs such as *mutt*, *pajeet* and *gypsy* (see Figure 2). In our ED dataset, explicitly antagonistic neologisms target other platforms and their users. One such neologism, *jewtube* (an antisemitic reference to YouTube), is used in similar contexts as a range of other terms referring to online platforms and websites, including *youtube*, *newgrounds*, *twitter*, *furaffinity*, *deviantart*, *facebook*, and *failbook*, and later also *twitch* and *instagram*. While past research has considered the role of anti-semitic vernacular in 4chan/pol/Tuters and Hagen (2020), ED can also be explicitly connected to this ideology, having been owned by convicted extremist Andrew Auernheimer (also known as weev)^6^ around the early 2010s JNS.org (2020), when our ED corpus begins.

Antagonistic terms like *beta*, *burger*, or *kebab*, which are based on shifting meanings of established words, can be deemed particularly significant because of their ambiguity for “outsiders” of the platforms. While such vernaculars may thus be understood to retain significance in unambiguously racist milieus, as memes they also simultaneously undergo a process of loss or reduction of their original meaning—known technically as “semantic bleaching” Sweetser (1988)—which is also key to the performative dimension of these vernaculars, as discussed below. Additionally, we can use the terms they have developed to empirically verify exactly what groups and actors are considered principal antagonists, as one possible implication of them having their own label.

### Ironic Word Play

Our analysis of the 4chan and ED data yields a set of words that speak directly to the ironic language games of both platforms. As noted earlier, a key characteristic of the subcultural communities of the deep vernacular web is their penchant for a deceitful, apparently ironic, yet decidedly harmful play with language. Previous authors have therefore characterized these ambiguous, irresponsible language games as the “masquerade (of) non-ironic Nazis” pretending to be “ironic Nazis” Goldenberg and Finkelstein, (2020), or, as Lewis (2020) has it, the expression of “actual-bigotry-camouflaged-as-ironic-bigotry”.

A key neologism that was identified in both our corpora as referring to this type of ironic language games, is *lulz*. At the time of writing, *lulz* for instance figures in the motto “In lulz we trust” displayed on the front page of ED. This term is derived from *lol*, an abbreviation of “laughing out loud” that can be traced back to the early days of the web and is used to represent laughter. On 4chan and ED, *lulz* has taken on the generic meaning of “those things that can be enjoyed by the community,” referring to utterances that appear entertaining, comical, fun, and ironic (see Uitermark, 2011 for the early uses of the term on 4chan). Following Whitney Phillips (as quoted in Lewis, (2020)), this “mystique (and) fun” of *lulz* is what renders these language games so ambiguous and therefore “ethically paralyzing.”

On a linguistic level, the neologism *lulz* is iconic for this type of language-play, as it is both an explicit label for, as well as performance of, an ironic language game. Its ties to the term *lol* can easily be recognized, yet by replacing the “o” with an “u” and by adding the conventionalized “z” as a marker of the plural form (similar to e.g. *internetz*, another term that can be found in our dataset, albeit less prominently), its original meaning is also rendered “ironic” and appropriated to the more radical online environments of 4chan and ED. As such, our analysis shows that *lulz* is part of a broader category of vernacular innovations that could be called “ironic wordplay.” A related example from the 4chan dataset is for instance *lel*, another variation of *lol*.

One subcategory of these misspellings consists of intentional and conventionalized misrepresentations of English words, rendering their meanings ironic. Mechanisms to this end that can be observed in our data include switching around characters, substituting characters, adding characters or combinations of these. Such mechanisms evoke typographical errors, and recall the alternative spellings, “leetspeak,” and abbreviations associated with game and internet memes such as LOLcats (Miltner, 2014). Examples of ironic misspellings in the ED dataset are *evar*, a mocking reference to *ever*, *rekt* for *wrecked*, *copypasta* to refer to blocks of text that are copy-pasted among many users Maloney et al. (2019), and *moar*, an intentional miswriting of *more* that figures in among others the *lurk moar* meme: an invitation to posters to internalize the conventions and games of the platform by reading and observing. Similar examples of vernacular corruptions of English words take on a radical nature, such as *raep* for *rape*.

**FIGURE 3:**
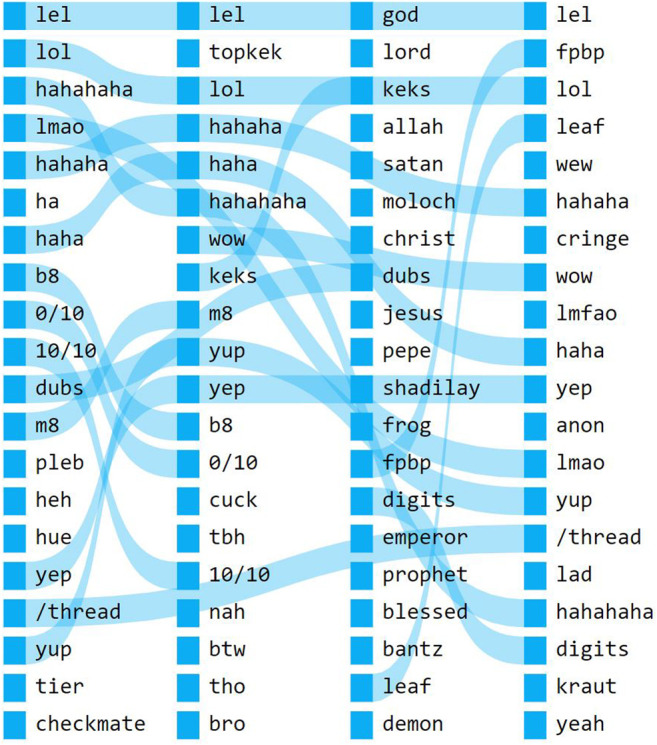
Rankflow diagram of *kek* in our 4chan dataset. Similar words are sorted vertically, from most similar to least similar. Each vertical stack represents a single snapshot, respectively 2012-2014, 2014-2016, 2016-2018, and 2018-2020.

Another subcategory of ironic misspellings comprises words for which the process of transformation from the original is more complex, to the point where any connection to English words is rendered opaque. These words are the product of more elaborate games that are explicitly tied to platform affordances. A key example is the term *kek*, which our analysis brings to the fore as one of the most dynamic words in the 4chan corpus (see Figure 3). While in the 2012-2014 and 2014-2016 snapshots the term is most similar to expressions of laughter such as “lol” and “haha,” the term is used in a strikingly different context in the 2016-2018 snapshot. In the latter, terms that are most similar to *kek* comprise mainly religious terminology, including words such as *god*, *lord*, allah, and *satan*, as well as references to the “Pepe the frog” meme, including the terms *pepe* and *frog*, all of which have been connected the emergence of the so-called “Cult of Kek” (Asprem, 2020). In the subsequent 2018-2020 snapshot, the term is again most similar to expressions of laughter.

While the fact that the term reverted from its quasi-religious context of use to an earlier one may seem to suggest a less robust innovation, here one should take into account subcultural dynamics. On 4chan, as in other subcultures, vernacular terms and practices lose their value when co-opted by the mainstream (de Zeeuw et al., 2020). Indeed the mainstreaming of the Cult of Kek as well as of “Kekistan”—which came to prominence on Youtube as a kind of imaginary homeland for trolls in 2017-18 (de Keulenaar, 2018)—should be understood as key ethnographic factor accounting for this last shift in meaning.

### Reflexive Performativity

A common theme in our data were words and phrases that were used to act-out self-reflexive in-group practices in dialogue with aspects of the infrastructures of the platforms that support this community. These practices may be considered as *performative* in reference to Austin [1975 (1963)]’s theory of ritualistic forms of speech. In the context of how this type of language is used on 4chan, such performative speech acts—or order words—can be understood as magically conjuring alternate realities (Asprem, 2020).

Long central to this community has been its self-mythologization through exploits and hoaxes perpetrated against the media or other “mainstream” institutions. Accounts of these exploits then feed back into the community’s self-understanding—as in the case of a Fox News affiliate once described 4chan as an “Internet Hate Machine” Phillips (2015)—which in turn become myths by which they understand themselves as a group^7^. The most well-known example of a performative practice is *shitposting*, which has in recent years cautiously entered popular vernacular as meaning “incoherent jokes, hasty Photoshopping, mashups, irrelevance, errors in spelling or grammar” Klee (2016), intended to inject vitriol into social media conversation.

An example of a performative practice explicitly connected to the affordances of 4chan is *trips*, short for *trips of truth*, a phrase related to the practice of attributing greater significance to posts on 4chan that are identified with a particular ID number. On 4chan, all posts are stored with a unique, sequential ID number; this number is displayed relatively prominently in the 4chan interface and is also used to refer to one post from another. Though a poster has no control over the number, particular combinations are attributed to a greater significance; these are IDs ending with two identical numbers (*dubs*); three numbers (*trips*); or four (*quads*). IDs that consist of only one repeating number are particularly rare and, accordingly, particularly significant; these are dubbed *GETs*.

While it would be naive to suggest that posts on 4chan with a significant ID number are “magically” regarded as more true than others, it is nevertheless common to reply to such posts with a phrase referencing *truth* (the phrase “trips of truth” alone occurs over 24,000 times in our dataset). In this “trips of truth” functions as a kind of folk theory about how technology works—a jocular vernacular affordance McVeigh-Schultz and Baym (2015) through which an understanding of how the platform works shapes the discourse occurring on it. More to the point, the term shows how the materiality of the platform can affect the discourse and notably the signification of particular parts of the discourse, as a seemingly random material factor like the ID number of a post can afford greater visibility not through explicit platform features but because it is reified into a marker of significance apropos of nothing.

A conceptually similar performative practice is the use of *powerword* on ED, where it refers to referencing someone by their real name rather than an online nickname—what in another context would be called *doxing*, which is considered a major violation and bannable offense in many forums including 4chan. The term *powerword* originates in Dungeons and Dragons role-playing games, as a synonym for magic spells. On ED, it mockingly positions using someone’s real name as such a “spell” is a well-known trope in folklore and mythology, as in the Old Testament when Adam names the beasts in the Book of Genesis. On ED, the practice is described more prosaically as such on the Powerword page:

“Most people believe that the relative anonymity of the net allows them to indulge their most perverted and unacceptable desires without consequence. However, since these same people are not elite hackers who have taken Computer Science III, they don’t realize how easy it is (...) to track down their dox and reveal all to the world.”

Indeed, a significant part of ED comprises pages about people. Many of these are about internet users or community members that have fallen out of favour with the ED for one reason or another, and here documenting their real name (or doxing) can be used as a way to intimidate or otherwise harass them. Reifying the practice as “using a powerword” seems to have given it a life of its own, however, and there are many pages where someone’s real name is linked to the Powerword page even if the name of the person in question is well-known^8^. The act of “exposing” someone’s real name has little significance on those pages, but nevertheless connecting any reference to a real name on ED to powerwords emphasizes how documenting someone’s personal details gives a certain type of power, in general if not in particular for someone whose personal details are already widely known. This arguably follows from the fact that ED is a wiki, a type of platform for which documenting facts is the primary purpose. On any wiki, knowledge is power; ED is only different in how it makes this explicit, reifying it in the narrow case of documenting someone’s real name, and then exaggerating this in a manner typical of the DVW.

The idea that performative practices can enact alternative realities is often referred to as *meme magic* within the community itself. The primary example of this is the unexpected election of Donald Trump as president of the United States in 2016; as Dale Beran (2017) notes, the community likes to claim that they “memed Trump into the White House.” More recently, meme magic has been positioned as responsible for the rise in value of various cryptocurrencies and the momentary but meteoric rise of the stock value of video game retailer GameStop (see Boyston et al., 2021). The emergence of this concept of *magic* is reflected in our data: in the 2012-2014 and 2014-2016 snapshots of our 4chan corpus, the term *magic* is used in the same context as words referring to satanism (e.g. *devil*, *lucifer*, *satan*), pagan religion (*odin*, *gods*), and conspiracies (*illuminati*). In particularly the 2016-2018 snapshot, the term"s context of use is more similar to that of words referring to memetics, including *dank*, *pepe*, *kek*, *frog*, and *memes*. In the 2018-2020 the predominant language game then again shifts to uses of *magic* similar to terms like *devil* and *satan*, which relates to the context of *kek*, as discussed above.

Crucially, these performative practices attribute a kind of magical significance to a feature of the platform, though in a deeply “ironic” register. In the case of trips of truth, 4chan’s attribution of a unique number to any given post; in the case of powerwords, the notion that “knowledge is power” that is built into a wiki. While it would be misleading to say that the users of these terms *actually believe* in the power ironically ascribed to these practices, the simple act of signification represented by giving these things a name positions them as *performative artefacts* around which the subculture imagines itself in a self-reflexive manner. Furthermore, these examples empirically demonstrate that ritualized subcultural expressions such as doxing (Phillips, 2015), can be detected at the level of language innovation.

## Discussion

Overall the data displays a rich vocabulary of self-reflective terms that reveal both the impact of the respective platforms’ materiality on its discourse as well as the preoccupations of its users. Emerging as they do from the “deep vernacular web,” it should also be noted that the terms discussed here represent a proverbial tip of the iceberg, as we limit ourselves to highly frequent words only. These practices reveal these sites as a self-reflective language community, highly aware of its own idiosyncratic use of language in a way that is ambiguously detached from actual beliefs or facts. This has a self-reinforcing effect, as in the case of a term like *powerword*, which seems to represent a kind of conceptual forking of doxing into an entire practice in dialogue with the affordances of ED as a wiki. In practically every case these practices are highly antagonistic, not only to the out-group but frequently, as in the case of *powerword*, to the supposed in-group as well. These examples reveal the obsessive preoccupation of this language community with turning contingent and dynamic processes into expressions, which then gain their “meme value” by popular uptake until they are deemed to be used too much by “normies” Literat and van den Berg (2019) after which their popularity typically drops.

Through the lens of pragmatics we can understand the ways in which *the media shape the meme*–to use the emic language of the meme so central to this community. While both 4chan and ED share a common ideology and broad discursive style, our findings also suggest different preoccupations—attributing significance to posts IDs in the case of 4chan (trips), and real user names in the case of ED (powerword). The former refers to the ephemeral affordances of 4chan while the latter speaks directly to the wiki affordance in which content is authored rather than being anonymous. Indeed, their language use is often entangled with the materiality of the infrastructures that host them as well as ideas on how these platforms *should* be used (Gershon, 2010). While many of these terms can be characterized as racist, antisemitic, homophobic, or otherwise harmful, this offensive language may also be understood as part of “an equilibrium of offense” Auerbach (2011), a community strategy intended to repel potentially unsympathetic site visitors—an observation which does not preclude also taking these terms’ meaning at face value as expressions of extreme, even genocidal levels of antagonism.

A particularly evocative example from our findings is the term *kek* (on 4chan), which arguably signifies the emergence of an entire coherent conceptual space (see Figure 3). Previous studies have shown that *kek* first appeared in the multiplayer game *World of Warcraft* (Burton, 2016; Wendling, 2018; Hermansson et al., 2020). In 2015-16 on/pol/the word spawned an online religion “situated somewhere between parody, make-believe, metapolitical strategy, genuine messianic expectations, and magic” (Asprem, 2020 23). Through a series of vernacular innovations, *kek* thus went from an expression of laughter to an ironic reference to a pseudo-deity, to a right-wing extremist trope. Borrowing a term from the fields of contemporary evolutionary theory—as opposed to the out-dated variety of memetics—we can conceptualize this as instance of “stigmergy,” whereby a “left-behind” form Steels, (2012) is used by the community as a point of alignment for new meanings and practices.

While anons appear to attribute quasi-magical properties to their performative speech acts consistent with the overarching ironic tone of the forum, this should not be taken to believe as an actual belief in magic so much as a playful appropriation of the “trappings of magic.” Asprem connects this with Discordianism, a parody religion popular in the Californian counterculture, that worshiped chaos and a parallel can also be drawn to post-modern “chaos magick,” in which symbols are used to achieve desired ends and without the practitioners necessarily professing inherent belief. From this perspective anons “actual” beliefs are thus less important than the fact that these speech acts are repeated over and over again.

From these three main aspects of 4chan and ED’s innovative language use, an impression emerges of a language community acutely aware of the fact that language is, if not actually magical, at least “meme-like,” in that it seems to have its own agency whether or not it speakers *actually* believe in its efficacy—as such we could say that the terms *kek* and *powerword*, while deeply ironic, nevertheless denote a core *performative* function of language in the deep vernacular web. Obfuscated by a thick layer of irony, the vernacular of the community reveals both their political preoccupations as well as an active engagement with the affordances of the specific platforms studied here. This then serves as a way to internally mark important practices and antagonisms as well as a method to playfully demarcate the community itself.

## Conclusion

This paper set out to study the meaning-making processes of the language community of which 4chan and ED are a part, which were examined through the lens of language games. While this community often discusses their own artefacts emically in terms of “memes,” we find it useful to understand their artefacts through the lense of linguistic pragmatics. As a heuristic for identifying and eliciting traces of these games from the large textual corpora mined from 4chan and ED, the research operationalized methods from natural language processing. For one thing, this concerned a method for neologism detection in order to identify emerging and idiosyncratic vernacular terms (that is, terms that are not in the standard English). For another, word embeddings were used to identify words marked by shifts in meaning over time, which potentially point towards newly emerging or changing language games.

These methods, which could be deemed “data hermeneutics” (see Romele et al. (2020)), are part of a larger cycle of interpretation, which comprises further close-readings and contextualization of the retrieved vernacular innovations—drawing among others from expert background knowledge in media theory and the study of online subcultures. On a methodological level, this paper has thus sought to contribute to the development of “quali-quantitative” approaches to big data (Venturini and Latour, 2010). More specifically, the method on offer in this paper can be transferred to the analysis of other corpora, facilitating a bottom-up approach to the detection of vernacular innovations that offers researchers a starting point for decoding and interpreting language games that can appear completely strange and alien to outsiders.

Previous work has investigated how antagonistic vernacular propagates from fringe media to mainstream media (see e.g. de Zeeuw et al., 2020). In contrast to such a cross-platform approach, the present paper investigated a limited set of dynamics internal to these groups pertaining to how such vernaculars appear and circulate within the fringe platforms of 4chan and ED. It is by identifying and decoding their language games that we are able to determine who or what is being antagonized by them. The argument here is that we need to understand the vernacular language games of antagonistic communities in their “subterranean” home environments before we can properly address their involvement in “above ground” cultural conflicts.

Previous research noted the centrality of game-life “laws” to online discussion forums such as 4chan—for example Poe’s Law, according to which no statements can be taken at face value. Such laws form the basis of the web-native subcultural worldview of the “deep vernacular web” which marked itself off from the mainstream in part by its fidelity to these rules (de Zeeuw and Tuters 2020). Our empirical analysis of language games does indeed confirm this hypothesis. Within these spaces, vernaculars are used consistent with Deleuze and Guattari’s post-structural pragmatics whereby every statement is connected to the implicit presupposition and social obligations of what, following Luc Steels’ pragmatics, we call a *subcultural language community*. Moreover, and in acknowledgment of pragmatics’ attention to context, we must also take into account the fundamental relationship between language games and platform affordances.

Finally, while this community is often identified as the source of memes online, our contention has been that memetics is a poor conceptual frame of analysis for understanding the manifold ways that these so-called memes are entangled with the community’s modes of existence. Multiple cases from our dataset—for example *trips*—problematize the *medium insensitivity* of memetics (Sampson, 2012). While the word embeddings and nearest neighbours of these vernaculars provide us with hints as to the meanings of these words, a complete interpretation also requires an affordance analysis of the discrete material contexts in which such terms originate and evolve—such as the ephemeral and anonymous structure of imageboards. Our quantitative assessment of the 4chan and ED corpora furthermore appears to and confirms the notion that ED is an *accumulative* archive whereas 4chan is the more *ephemeral* site where that culture is performed and enacted. 4chan can be characterized as an ephemeral, anonymous imageboard where posts are only preserved a limited amount of time, affording rapid evolution and “forgetting.” Encyclopaedia Dramatica, on the other hand, is a wiki that specifically seeks to archive subcultural artifacts and knowledge so that they are not forgotten.

ED and 4chan can then be positioned as part of a common subcultural language community, sharing preoccupations and styles but also differing significantly in their materiality, which is reflected in the fact that their prominent vernacular styles only partially overlap. What is common is their playful and innovative use of language, prominent deployment of irony and the hyper-antagonistic, strongly racist patterns clearly visible in this practice.

## Data Availability

The datasets presented in this study can be found in online repositories. The names of the repository/repositories and accession number(s) can be found below;https://zenodo.org/record/4792071.

## Footnotes

1 Venturini, T. (2003). A Sociology of Online Monsters: Online Conspiracy Theories and the Secondary Orality of Digital Platforms. Submitted Theor. Cult. Soc. manuscript in review.

2 As a result, there have been episodes during which multiple sites claimed to be the “real” Encyclopedia Dramatica. At the time of writing, at least two such sites exist, one at encyclopediadramatica.online and one at encyclopediadramatica.wiki. Both comprise, to a large extent, the same historical data. The corpus used in this article was sourced from the latter.

3 Using Gensim’s implementation of Word2Vec for Python, with vector size 100, alpha 0.025, window 5, sample 0.001, min frequency 5, negative sampling 5

4
https://storage.googleapis.com/books/ngrams/books/datasetsv3.html, version 20120701

5 The meaning of this term can be traced back to an antisemitic image created originally by the pseudonymous cartoonist A. Wyatt Mann in a “satirical” comic book about holocaust denial, which would go on to become uses as a meme referred to as the “happy merchant” (Bernstein, 2015).

6 While Auernheimer had previously been one of the key informants in an earlier ethnographic study of 4chan Coleman (2014), by the mid 2010s he had become co-manager of the Neo-Nazi website *The Daily Stormer*.

7 On 4chan, the performative processes by which an otherwise relatively anonymous group of strangers comes to perceive a coherent sense of in-group identity through a dialogue with representations of themselves in the media has been called “weird collectivity” (Hagen and Tuters 2021). Notably, these self-referential and performative qualities had been observed in a previous study of the “Anonymous” hacker movement, which had used 4chan as one of its primary sites of coordination online (Uitermark, 2017)

8 e.g. for animator Jonti Picking (also known as Weebl), memetic Eurovision Song Contest participant Sergey Stepanov (Epic Sax Guy) or biblical prophet Moshe Rabbenu (Moses).
